# ACCEPTABILITY OF HIGH-INTENSITY FUNCTIONAL STRENGTH TRAINING AT HOME FOR POSTINJURY OLDER ADULTS

**DOI:** 10.1093/geroni/igad104.2310

**Published:** 2023-12-21

**Authors:** Ashley Morgan, Jennifer Heisz, Ada Tang, Lehana Thabane, Julie Richardson

**Affiliations:** McMaster University, Hamilton, Ontario, Canada; McMaster University, Hamilton, Ontario, Canada; McMaster University, Hamilton, Ontario, Canada; McMaster University, Hamilton, Ontario, Canada; McMaster University, Hamilton, Ontario, Canada

## Abstract

Regular exercise plays a vital role in optimizing aging and preventing functional decline. Despite the recognized benefit of exercise, adherence is challenging, and acceptability of exercise interventions is crucial. Acceptability is influenced by various factors, including content, context, and delivery. The purpose of this qualitative descriptive study is to determine the acceptability to older adult participants of a home-based high-intensity functional strength training (HIFST) intervention delivered by a physiotherapist via videoconferencing. HIFST involves the use of periods of ‘hard’ effort using everyday strength-building movements alternating with ‘easy’ recovery periods. This study accompanies an ongoing pilot randomized controlled trial investigating the feasibility of HIFST (completion: spring 2023). We are conducting semi-structured interviews and a short anonymous survey with participants who have completed HIFST. Data collected is being analyzed using qualitative content analysis. To date, results from 8 participants (2 men, 6 women, 55-81 years) demonstrate a positive overall impression of HIFST with all participants noting enjoyment, a good experience, and/or meeting their expectations. Participants reported certain aspects less/more enjoyable than others (e.g., specific exercises). No technical difficulties have been noted and many participants appreciated the convenience of an online format. Prior knowledge of interval-based exercise was minimal for most participants and the majority commented on a preference for shorter intervals with shorter rest periods. Further data collection and analysis will provide a contextualized understanding of the HIFST intervention and important considerations for exercise prescription in an aging population.

